# The Possibility of IPC to Prevent Ischemic-Reperfusion Injury in Skeletal Muscle in a Rat

**DOI:** 10.3390/jcm12041501

**Published:** 2023-02-14

**Authors:** Takanori Morikawa, Miyako Shimasaki, Toru Ichiseki, Shusuke Ueda, Yoshimichi Ueda, Kan Takahashi

**Affiliations:** 1 Department of Anesthesiology, Kanazawa Medical University, Daigaku 1-1, Uchinada, Kahoku-gun, Ishikawa 920-0293, Japan; 2 Department of Pathology 2, Kanazawa Medical University, Daigaku 1-1, Uchinada, Kahoku-gun, Ishikawa 920-0293, Japan; 3 Department of Orthopaedic Surgery, Kanazawa Medical University, Daigaku 1-1, Uchinada-machi, Kahoku-gun, Ishikawa 920-0293, Japan; 4 Department of Pathology, Keiju Medical Center, 94, Tomiokamachi, Nanao, Ishikawa 926-0816, Japan

**Keywords:** ischemic preconditioning, reperfusion injury, apoptosis, vascular endothelial growth factor, cyclooxygenase 2, 8-hydroxyguanosine

## Abstract

Blood removal with air tourniquets for a long time induces muscle damage after reperfusion. Ischemic preconditioning (IPC) has a protective effect against ischemia-reperfusion injury in striated muscle and myocardium. However, the mechanism of action of IPC on skeletal muscle injury is unclear. Thus, this study aimed to investigate the effect of IPC in reducing skeletal muscle damage caused by ischemia-reperfusion injury. The hindlimbs of 6-month-old rats were wounded with air tourniquets at a carminative blood pressure of 300 mmHg on the thighs. Rats were divided into the IPC (−) group and the IPC (+) group. The vascular endothelial growth factor (VEGF), 8-hydroxyguanosine (8-OHdG), and cyclooxygenase 2 (COX-2) were investigated by protein levels. Quantitative analysis of apoptosis was performed using the TUNEL method. Compared with the IPC (−) group, the IPC (+) group retained the VEGF expression, and the COX-2 and 8-OHdG expressions were suppressed. The proportion of apoptosis cells decreased in the IPC (+) group compared with the IPC (−) group. IPC in skeletal muscles proliferated VEGF and suppressed inflammatory response and oxidative DNA damage. IPC has the potential to reduce muscle damage after ischemia-reperfusion.

## 1. Introduction

Air tourniquets are generally used to prevent excessive bleeding while ensuring a bleeding-free surgical field in limb surgeries. However, prolonged use of air tourniquets may cause serious complications, such as rhabdomyolysis, compartment syndrome, apoptosis due to reperfusion disorders, and thrombosis. The same is true for skeletal muscles, where irreversible injury occurs 3 h after ischemia, and completely irreversible muscle damage occurs after 6 h [1]. These factors include the fact that oxidative stress induced by ischemia-reperfusion injury causes glycogen synthase kinase-β (GSK-3β) to migrate from the cytosol to the mitochondria and directly bind to the mitochondrial permeability transition pore (mPTP) complex, and that the mitochondrial translocation of GSK-3β enhances cytotoxic reactive oxygen species (ROS) production in mitochondria [2]. ROS also opens the mPTP, causing morphological expansion, collapse, and loss of mitochondrial function. As a result of these factors, insufficient energy production in mitochondria, disruption of intracellular calcium concentration, homeostasis at the plasma membrane and endoplasmic reticulum, and apoptosis and necrosis due to ischemia-reperfusion injury are considered to be important mechanisms. In addition, ROS leads to decreased production of NO, which has vasodilatory, platelet-aggregation inhibitory, and leukocyte-adhesion inhibitory effects, and decreased NO production reduces tissue blood flow. Furthermore, impaired cells have been reported to release proinflammatory cytokines from microglia/macrophages [3]. These vicious cycles are thought to aggravate ischemia-reperfusion injury.

Based on the concept of reducing ischemia-reperfusion injury to prevent irreversible tissue injury, the effect of ischemic preconditioning (IPC) has been examined, and its effectiveness has been reported. IPC is a phenomenon in which resistance to subsequent long-term ischemia is enhanced by a preceding short period of ischemia and was first reported by Murry et al. in 1986 [4]. The beneficial effects of IPC in ischemic heart disease and cerebrovascular disease have been reported to lead to a reduction in tissue damage by promoting vascular growth factor expression, attenuating oxidative stress, and reducing the inflammatory response of tissues [5].

There are few reports on the mechanism of IPC for the prevention of skeletal muscle injury. IPC with three cycles of 5 min and IPC with three cycles of 10 min have been reported. Their results have shown that IPC reduces skeletal muscle damage [6,7,8]. Additionally, many of the previous reports have examined the condition immediately after ischemia, but when considering postoperative rehabilitation and pain in actual clinical practice, it is necessary to examine the temporal changes from ischemia-reperfusion as well. Therefore, when determining the efficacy of IPC, in addition to assessing cell death and oxidative injury, local inflammation and muscle fiber diameter should also be considered.

Therefore, this study aimed to investigate the inhibitory effect and mechanism of skeletal muscle injury by IPC using a rat model that induces ischemia-reperfusion injury in the gastrocnemius muscle of the lower leg by air tourniquet wrapped around the hindlimb thigh.

## 2. Materials and Methods

### 2.1. Animal

This study was conducted in accordance with the Kanazawa Medical University Guidelines for Animal Experimentation. Male Sprague–Dawley rats aged 6 months (*n* = 45) (Sankyo Lab Service, Tokyo, Japan) were used for the experiment. This study was conducted in accordance with all guidelines of the Animal Research Committee of Kanazawa Medical University (#2021-35).

### 2.2. Hindlimb Ischemia Model

All animals were weighed before the experiment. They were anesthetized by intraperitoneal injection of 0.3 mg/kg medetomidine hydrochloride, 2 mg/kg midazolam, and 2.5 mg/kg butorphanol. Then, blood pressure was measured in the tail artery of the rats using a non-invasive sphygmomanometer (BP-98AL, Softron, Tokyo, Japan) (*n* = 5). Blood pressure at six months was 117 ± 9 mmHg. In this study, the air tourniquet pressure was stipulated as 150 mmHg or more than the systolic pressure, so the carminative pressure was 300 mmHg in all rats, and the ligation site was unified to the left hindlimb thigh. Blood pressure was measured every hour during blood purification with no significant change during the course. During anesthesia, a thermocouple temperature sensor (BWT-100A, RET-2, BRC Bioresearch Center, Nagoya, Japan) was inserted into the rat rectum to reduce the rectal temperature to 38 ± 1 °C, and a heating pad (BWT-100A, BRC Bioresearch Center, Nagoya, Japan) was used to maintain this temperature.

**Figure 1:**
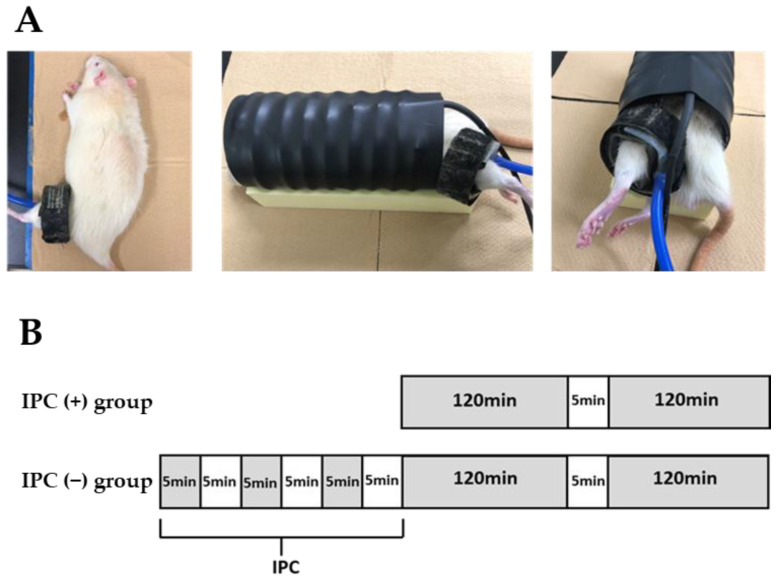
Animal model of ischemia and reperfusion. (**A**) A tourniquet cuff designed for a human finger applied to the proximal portion of the rat hindlimbs. (**B**) Experimental protocol. In the IPC (−) group, a tourniquet was applied for 120 min (black square: 300 mm Hg) twice, with a 5 min reperfusion period (white square) in between. In the IPC (+) group, 5 min of ischemia (300 mmHg) and 5 min of reperfusion were repeated three times and followed by the protocol used in the IPC (−) group. In both groups, the gastrocnemius muscle was excised under general anesthesia 72 h later for further experimental investigation.

A tourniquet cuff (width 2.5 cm, 20-64-700, VBM, Sulz am Neckar, Germany) for human fingers was wrapped around the thigh of the left hindlimbs of the rats (Figure 1A) using the tourniquet device (Digital Tourniquet 9000, VBM, Sulz am Neckar, Germany). The rats were divided into two groups (IPC (−) and IPC (+) groups). In the IPC (−) group (*n =* 32), blood was demolished for 120 min, reperfusion was performed for 5 min, and ligation was added for 120 min before reperfusion (Figure 1B). In the IPC (+) group (*n* = 32), the first 120 min of ligation was preceded by three loads of 5 min ligation and 5 min reperfusion. The ischemic gap time was selected to be the same as the IPC reperfusion time. Furthermore, in 6-month-old rats (*n* = 8), simply wrapping the tourniquet around the hindlimb thigh was enough to wrap the tourniquet around the IPC (−) group and the IPC (+). A sham group was established with the same time lapse, but not ischemia (the sham group). In the IPC (−) and IPC (+) groups, the gastrocnemius muscle of the left lower leg was removed immediately 24, 48, and 72 h after reperfusion (*n* = 8 each).

As a preliminary experiment, 6-month-old rats were subjected to the same ligation as the IPC (−) group. At 48 and 72 h after reperfusion (*n* = 8 each), the gastrocnemius muscle of the left lower leg was removed under general anesthesia, and after formalin fixation, hematoxylin-eosin (HE) was stained. As a result, edema was observed in the muscle tissue at 48 h after reperfusion and tissue enlargement, but after 72 h, tissue edema improved at all age periods. Separately, the 6-month-old rats were subjected to the same ligation as the IPC (−) group, reperfusion was performed at 0, 24, 48, and 72 h, the gastrocnemius muscle of the left lower leg was removed under general anesthesia, formalin fixation was performed, and TUNEL staining was performed (*n* = 8 each time). As a result, the ratio of TUNEL-positive nuclei to the total number of nuclei was 1.2 ± 0.3% at 24 h after reperfusion, 2.1 ± 0.2% at 48 h after reperfusion, and 2.0 ± 0.2% at 72 h after reperfusion. The number of TUNEL-positive nuclei increased at 48 and 72 h after reperfusion compared with 24 h after reperfusion. Therefore, the injury degrees in the gastrocnemius muscle of the left lower leg 72 h after reperfusion were compared.

### 2.3. Histology

Rats were divided into the IPC (−) group, the IPC (+) group, and the sham group (*n* = 8 each). At 72 h after reperfusion, medetomidine hydrochloride (0.3 mg/kg), midazolam (2 mg/kg), and butorphanol (2.5 mg/kg) were administered intraperitoneally. The gastrocnemius muscles were extracted soon after anesthesia. The extracts were immediately fixed with formaldehyde (Sigma, Aldrich Co, St. Louis, MO, USA), cross-sectional sections were created, and they were stained with HE. The cross-sectional sections of the gastrocnemius muscle were observed by a digital microscope camera (ABX53, Olympus, Tokyo, Japan). The edges of the collected sections were excluded during the evaluation. Additionally, 10 muscle fiber diameters were measured for each specimen, and the average value was obtained. The short cross-sectional diameter was used for the muscle fiber diameter because the measurement error is small with respect to the long axis of muscle tissue.

### 2.4. Immunohistochemical Study

The effect of IPC on skeletal muscle damage due to ischemia-reperfusion injury was studied using immunohistochemical staining of neovascular factors (VEGF), oxidative DNA damage (8-hydroxyguanosine (8-OHdG)), and inflammatory markers (cyclooxygenase 2 (COX-2)). In all groups, femoral sections were prepared, and sections obtained from the femoral proximal medial diaphysis of each group were deparaffinized with xylene and ethanol. For antigen retrieval, the sections were autoclaved at 121 °C for 15 min. Using 0.3% H_2_O_2_, endogenous peroxidase was eliminated, blocking was performed using a mouse or goat normal serum, and the primary antibody was made to react. As the primary antibody, anti-Bax rabbit polyclonal antibody (Abcam, Cambridge, UK), anti-VEGF rabbit polyclonal antibody (Proteintech, Rosemont, IL, USA), anti-8-OHdG mouse monoclonal antibody (Abcam), and COX-2 rabbit polyclonal antibody (Proteintech) were used at 1.25, 5.0, 20.0, and 5.0 µg/mL, respectively. The reaction time was overnight at 4 °C in a cool dark room. After reacting with the primary antibody, the secondary antibody (biotin) reacted with an enzymatic agent (streptavidin). After 5 min immersion in DAB to allow for color development, the nuclei were stained. Photographs were taken using a BX53 microscope (Olympus, Tokyo, Japan) and a DP71 camera (Olympus).

VEGF and COX-2 were randomly extracted from their respective muscle tissues with 10 fields of view (400 times) and quantified using ImageJ. In 8-OHdG, cell nuclei expressed by 8-OHdG were extracted from 10 fields of view per sample, and the IPC (+) group was compared with the IPC (−) group.

### 2.5. TUNEL Assay

Apoptosis detection kits (APOPTAG, Merck, Burlington, VT, USA) were used for the TUNEL method. Paraffin-fixed sections were deparaffinized, hydrophilically treated, treated with proteinase K for 15 min, and washed with PBS-Tween 20. 3% hydrogen peroxide was added, and the reaction was carried out at room temperature for 10 min to inactivate the endogenous substance. After reacting with equilibration buffer for 10 min, TdT enzyme was added and incubated at 37 °C for 1 h. It reacted with stop/wash buffer for 10 min and washed with PBS-Tween 20. The labeled antibody anti-digoxigenin conjugate was added and reacted at room temperature for 30 min. DAB was added, reacted for 2 min, and washed with distilled water. The counterstain was stained with Gil’s hematoxylin. The number of apoptoses was assessed by the ratio of the number of TUNEL-positive nuclei in one field of view to the total number of nuclei. A total of 50 fields of view were observed in one sample.

### 2.6. Statistical Analysis

The results were shown as mean ± standard deviation. The analysis of the difference in the mean value of the TUNEL-positive nuclear ratio, 8-OHdG-positive nuclear ratio, VEGF, and COX-2 expression area in skeletal muscle between the IPC (−) and IPC (+) groups was performed using Student’s *t*-test. *p* < 0.05 indicated a significant difference.

## 3. Results

### 3.1. HE Staining

**Figure 2:**
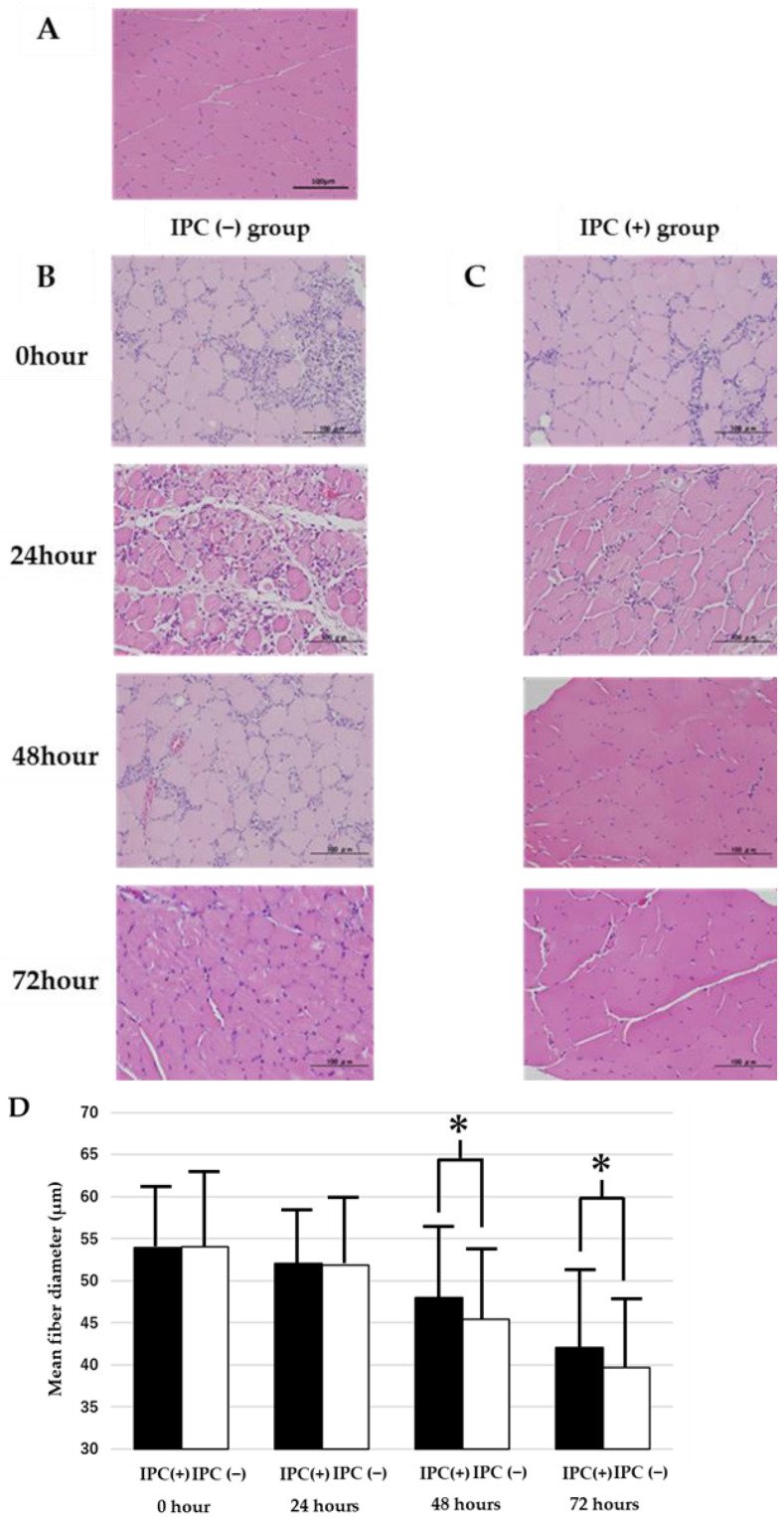
HE staining of gastrocnemius muscles was performed at 0, 24, 48, and 72 h after reperfusion. (**A**) Rats in the sham group. Muscle fibers were polygonal in shape, and individual muscle fibers were closely connected. (**B**) Rats in the IPC (−) group. Atrophic muscle fibers were present, but cytoplasmic destruction and polymorphonuclear leukocyte infiltration were absent. (**C**) Rats in the IPC (+) group. Histological results were similar to those shown in B. (**D**) The IPC (+) (block square) and IPC (−) groups (white square) are muscle fiber diameters immediately after 24, 48, and 72 h after ischemia in rat gastrocnemius muscle. Each group *n* = 8. Values are means ± standard deviation (* *p* < 0.05). Scale bar: 100 µm.

The histological findings on HE staining of the skeletal muscles at 0, 24, 48, and 72 h after reperfusion were compared among the sham, IPC (−), and IPC (+) groups (Figure 2).

In the sham group, the muscle fibers were polygon-shaped and in close contact with each other, and no difference in the size or atrophy of the muscle fibers was observed (Figure 2A). Immediately after reperfusion, polymorphonuclear leukocyte infiltration and degeneration were observed in some muscle fibers in both the IPC (−) and IPC (+) groups.

The muscle fiber diameters immediately after ischemia did not differ between the IPC (−) and IPC (+) groups, 54 ± 8.5 μm and 52 ± 8.4 μm, respectively. In the skeletal muscles 24 h after reperfusion, marked polymorphonuclear leukocyte infiltration and degeneration were observed in the IPC (−) and IPC (+) groups. The skeletal muscle fiber diameters after 24 h of ischemia also did not differ between the IPC (−) and IPC (+) groups, 52 ± 8.4 µm and 54 ± 8.5 μm, respectively. In the tissue 48 h after reperfusion, polymorphonuclear leukocyte infiltration was observed in some muscle fibers. However, most muscle fibers improved compared with the tissue 24 h after reperfusion. At 48 h after reperfusion, muscle fiber atrophy and a difference in the size of muscle fibers were observed in both the IPC (−) and IPC (+) groups. At 48 h after reperfusion, the skeletal muscle fiber diameters in the IPC (+) and IPC (−) groups were 48 ± 7.9 µm and 45 ± 8.8 µm, respectively (*p* < 0.05). At 72 h after reperfusion, extensive muscle fiber atrophy and small angular fibers were observed in the skeletal muscle tissue. At 72 h after reperfusion, the skeletal muscle fiber diameters of the IPC (+) and IPC (−) groups were 42 ± 9.0 µm and 35 ± 8.1 µm, respectively (*p* < 0.05). Furthermore, the myocyte morphology was maintained immediately after ischemia until 72 h, and no findings suggestive of cytoplasmic decay and necrosis were observed (Figure 2B,C). The muscle fiber diameters in the IPC (−) and IPC (+) groups immediately after reperfusion until 72 h after reperfusion were compared (Figure 2D). No difference in the muscle fiber diameter was observed between the IPC (−) and IPC (+) groups immediately after reperfusion until 24 h after reperfusion. At 48 and 72 h after reperfusion, the IPC (+) group exhibited mild muscle fiber atrophy compared with the IPC (−) group (*p* < 0.05).

### 3.2. TUNEL Staining

**Figure 3:**
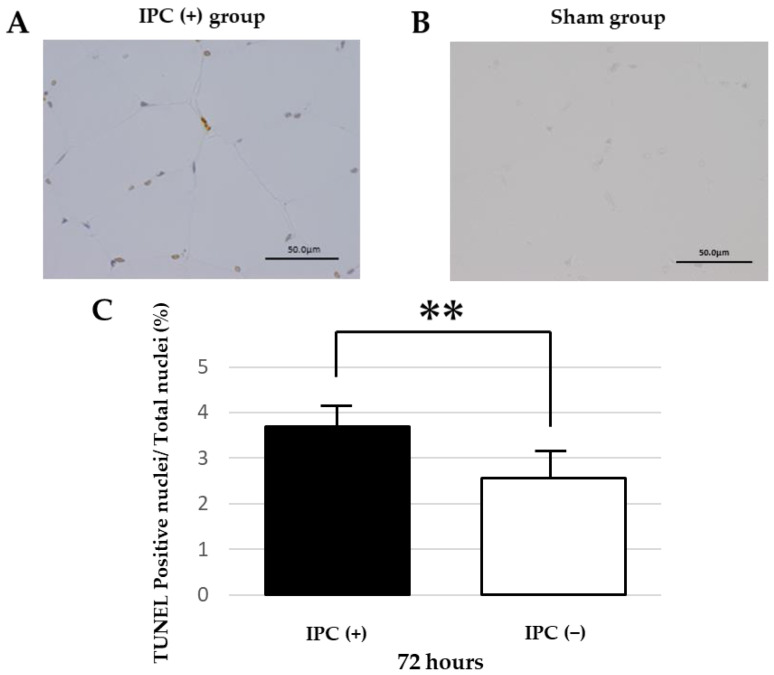
Apoptosis in rat gastrocnemius muscle tissue in ischemia-reperfusion injury. (**A**) TUNEL-stained gastrocnemius muscle of rats in the IPC (−) group 72 h after ischemia-reperfusion injury. DNA fragmented by apoptosis is stained brown (TUNEL-positive cells). Cells with unfragmented DNA are stained purple. (**B**) TUNEL staining of the gastrocnemius muscles of 6-month-old rats in the sham group. TUNEL-positive nuclei were not identified. (**C**) Ratios of TUNEL-positive cells to total cells in the gastrocnemius muscle of rats in the IPC (+) (black square) and IPC (−) groups (white square) at 72 h. Values are means ± standard deviation (** *p* < 0.01). Scale bar: 50 µm.

A comparison was conducted between the sham, IPC (−), and IPC (+) groups in DNA fragmentation that arises during the process of apoptosis using TUNEL staining. In the sham group, no TUNEL-positive nuclei were observed in the skeletal muscle (Figure 3A). In the IPC (−) and IPC (+) groups, TUNEL-positive nuclei were observed in some skeletal muscle at 48 and 72 h after reperfusion (Figure 3B). In a preliminary experiment using the IPC (−) group, no difference was observed upon comparing the number of TUNEL-positive nuclei at 48 and 72 h after reperfusion. Therefore, we decided to use the skeletal muscle tissue of 72 h after reperfusion and compared the ratio of the number of TUNEL-positive nuclei to the total number of nuclei between the IPC (−) group and IPC (+) group (Figure 3C). As a result, in the histological findings at 72 h after reperfusion, the IPC (+) group had a smaller proportion of muscle cells in which apoptosis had occurred than the IPC (−) group. However, no significant difference was observed (*p* = 0.026).

### 3.3. Immunostaining

**Figure 4:**
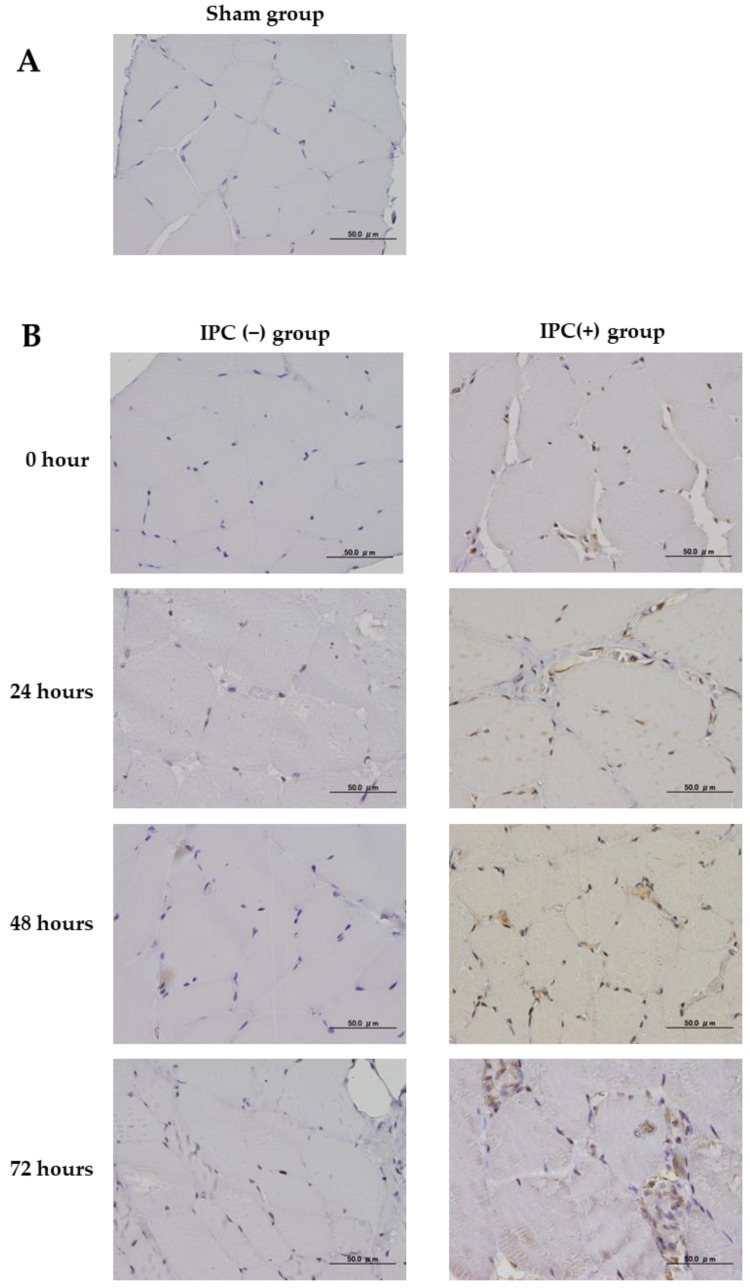
Immunohistochemical study of VEGF expression in the gastrocnemius muscle of rats. (**A**) The sham group. Scale bar: 50 µm. (**B**) Representative tissue findings immediately after and 24, 48, and 72 h after ischemia in the IPC (+) (left side) and IPC (−) groups (right side) are shown. Scale bar: 50 µm. In the IPC (+) (left side) and IPC (−) groups (right side), typical histological findings immediately after ischemia and then at 24, 48, and 72 h after ischemia are presented.

The vascular endothelial growth factor (VEGF) protein expression was analyzed immediately after reperfusion and at 24, 48, and 72 h after reperfusion by immunostaining according to IPC. The IPC (−) and IPC (+) groups were compared immediately after reperfusion until 72 h after reperfusion. In the IPC (+) group, VEGF expression was maintained in the vascular endothelial cells and around blood vessels in the tissue immediately after reperfusion until 72 h after reperfusion compared with the IPC (−) group (Figure 4A,B).

**Figure 5:**
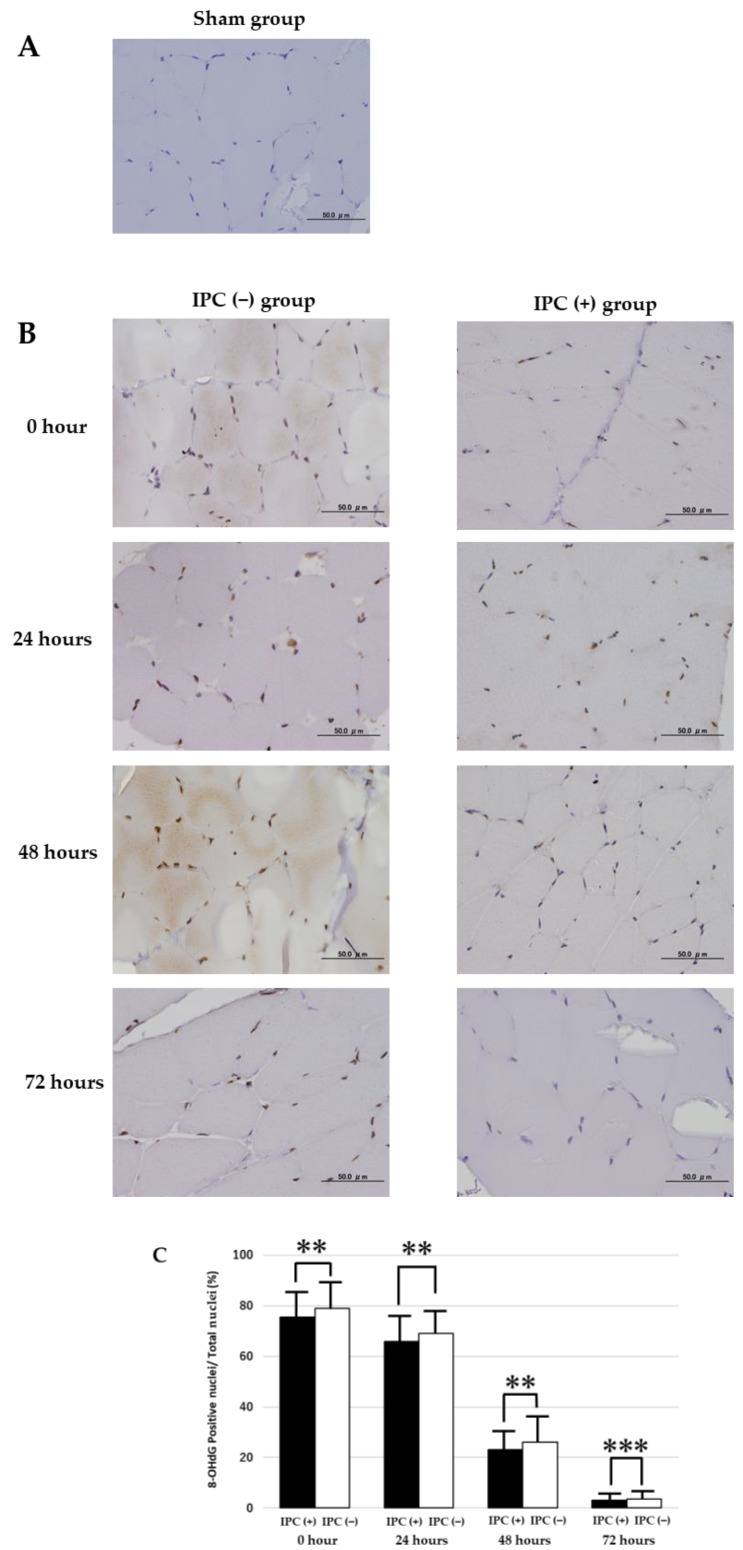
Immunohistochemical study of 8-OhdG expression in the gastrocnemius muscle of rats. (**A**) The sham group. Scale bar: 50 µm. (**B**) Representative tissue findings immediately 24, 48, and 72 h after ischemia in the IPC (+) (left side) and IPC (−) groups (right side) are shown. Scale bar: 50 µm. In the IPC (+) (left side) and IPC (−) groups (right side), typical histological findings immediately after ischemia and then at 24, 48, and 72 h after ischemia are presented. (**C**) Quantitative analysis based on the ratio of 8-OhdG expression-positive nuclei in the IPC (+) (black square) and IPC (−) groups (white square). The ratio of 8-OhdG-positive nuclei at 0, 24, 48, and 72 h is shown. Each group *n* = 8. Values are means ± standard deviation. Quantitative analysis based on the ratio of nuclei positive for 8-OhdG expression in the IPC (+) (black square) and IPC (−) groups (white square). The ratio of nuclei positive for 8-OhdG expression at 0, 24, 48, and 72 h is presented. Each group *n* = 8. Values are means ± standard deviation (** *p* < 0.01, and *** *p* < 0.001).

The analysis of 8-OHdG expression was performed in the sham group, the IPC (−) group, and the IPC (+) group immediately after reperfusion and at 24, 48, and 72 h after reperfusion by immunostaining. No 8-OhdG expression was observed in the sham group (Figure 5A). 8-OhdG expression was observed in the nuclei of muscle cells immediately after reperfusion until 72 h after reperfusion in the IPC (−) group and IPC (+) group (Figure 5B). Furthermore, 8-OhdG expression decreased over time immediately after reperfusion until 72 h after reperfusion. In the IPC (−) group and IPC (+) group, we compared the ratio of the number 8-OhdG-positive nuclei to the total number of nuclei immediately after reperfusion until 72 h after reperfusion. Compared with the IPC (−) group, the ratio of the number of 8-OhdG-positive cells in the IPC (+) group was decreased (*p* < 0.05) (Figure 5C).

**Figure 6:**
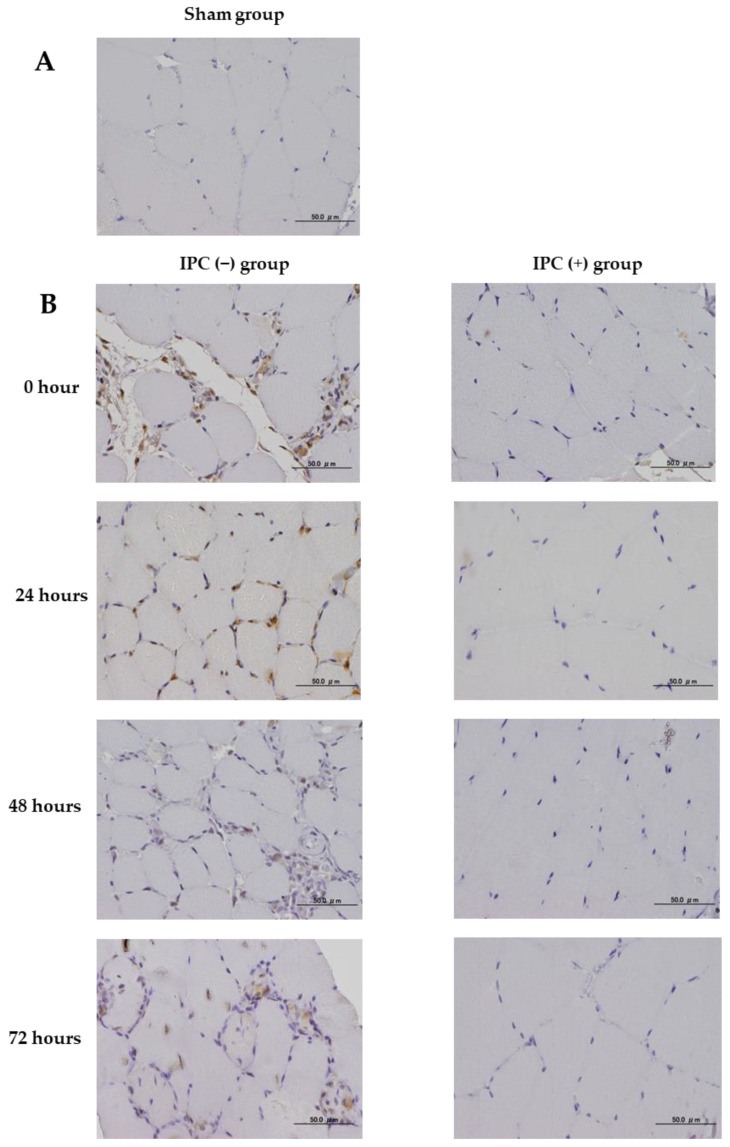
Immunohistochemical study of COX-2 expression in the gastrocnemius muscle of rats. (**A**) The sham group. Scale bar: 50 µm. (**B**) Representative tissue findings immediately after and 24, 48, and 72 h after ischemia in the IPC (+) (left side) and IPC (−) groups (right side) are shown. Scale bar: 50 µm. In the IPC (+) (left side) and IPC (−) groups (right side), histological findings immediately after ischemia and then at 24, 48, and 72 h after ischemia are presented.

The Inflammatory response was evaluated by conducting an examination using an analysis of COX-2 protein expression immediately after reperfusion and at 24, 48, and 72 h after reperfusion. No COX-2 expression was observed in the sham group (Figure 6A). COX-2 expression was observed immediately after reperfusion and 24 h after reperfusion in the IPC (+) group. However, no COX-2 expression was observed 48 and 72 h after reperfusion. In the IPC (−) group, 8-oHdG expression was observed in the muscle cell nuclei immediately after reperfusion until 72 h after reperfusion. In the IPC (+) group, 8-oHdG expression was inhibited immediately after ischemia until 72 after ischemia compared with the IPC (−) group (Figure 6B).

## 4. Discussion

Tourniquet in surgery generally causes irreversible changes in skeletal muscle due to prolonged ischemia. The preventive methods are eagerly desired. IPC has been reported to prevent and improve tissue damage in striated muscles and myocardium. In this study, we investigated the effects of IPC on skeletal muscle damage caused by ischemia-reperfusion injury using VEGF, oxidative DNA damage such as 8-OHdG, and inflammatory markers such as COX-2 and pro-apoptosis factors.

Blood ischemic time and reperfusion time during prolonged ischemia are considered risk factors for tissue damage [1,9,10]. Gürke et al. reported that using a tourniquet to load rat skeletal muscle with three cycles of IPC for 5 min suppressed skeletal muscle decline due to ischemia-reperfusion injury [6]. Therefore, we decided to repeat the IPC setting for three cycles of ischemia for 5 min and reperfusion for 5 min. Air tourniquets are often used during surgery at twice the systolic pressure or 200 mmHg above the systolic pressure, and the systolic blood pressure of the Sprague–Dawley rats was 100 mmHg. Since it was before and after, the ligation blood pressure was set at 300 mmHg in this study. Additionally, in actual clinical practice, ischemia is often canceled once in about 2 h when the ligation release time is 2 h or more to reduce the adverse effects of ligation. Thus, in this study, assuming a situation where the ischemic duration is long, 2 h of ligation was repeated twice with a 5 min reperfusion time. Cell death in a skeletal muscle may cause its functional impairment, making recovery of the atrophied muscle difficult. Therefore, we first examined apoptosis with and without IPC.

We confirmed the suppression of skeletal muscle damage by IPC and examined factors that may be related to muscle damage and suppression. These results showed that the IPC (+) group significantly suppressed apoptosis in skeletal muscle compared with the IPC (−) group in rat skeletal muscle 72 h after ischemia-reperfusion. Moreover, in the IPC (+) group, skeletal muscle atrophy was inhibited after ischemia-reperfusion compared with the IPC (−) group. This suggests that IPC may reduce skeletal muscle damage associated with ischemia-reperfusion. It has also been reported that oxidative stress occurs in ischemia and reperfusion injury, causing cellular oxidative DNA damage [11]. Therefore, in this study, 8-OHdG, which is used as an indicator of oxidative DNA damage in tissues, was examined [12]. We found increased 8-OHdG expression for oxidative DNA damage, which is known to induce various cell deaths, in the skeletal muscle of the IPC (−) group. In skeletal muscle of the IPC (−) group, increased 8-OHdG expression was observed. However, it was confirmed that the 8-OHdG expression was suppressed in the IPC (+) group. As an action in the striated muscle and myocardium of IPC, in open mitochondrial ATP-dependent K^+^ channels, IPC may suppress mPTP aperture by suppressing calcium overload [13] and induce anti-oxidative action [14]. These results showed that IPC could suppress the occurrence of oxidative DNA damage and reduce skeletal muscle tissue damage.

Next, we examined VEGF, a factor that generally inhibits ischemia-reperfusion injury; it is a cell growth factor that is expressed upon tissue ischemia and plays an important role in angiogenesis and improving blood flow via formation of vessels and extension of branches from existing vessels. The expression of VEGF on IPC was reported by Yoo et al. [15]. In this study, the IPC (+) group showed vascular growth factor expression immediately after IPC. The vascular growth factor expression by IPC was thought to be due to the effect of oxygen supply by angiogenesis to reduce ischemic tissue injury. However, in the sham and IPC (−) groups, the VEGF expression was only scattered and clearly less than that in the IPC (+) group. Physical damage, such as hypertension, smoking, and ischemia-reperfusion injury, damages the vascular endothelium and reduces the VEGF expression, and it has been shown that the vascular endothelium is injured by ischemia for 120 min [16]. The same mechanism was considered the reason for the poor VEGF expression in the IPC (−) group in this study. IPC pretreatment showed a significant increase in VEGF expression, indicating the possibility of repairing and preventing tissue damage by IPC preconditioning. Furthermore, in this study, IPC inhibited skeletal muscle atrophy following ischemia-reperfusion. The results of this study indicate that cell death and DNA oxidative damage associated with prolonged hemostasis can be suppressed by IPC, and VEGF expression may be involved, but the degree of skeletal muscle atrophy with and without IPC needs to be further examined. The present results showed atrophy of skeletal muscle fiber diameter after ischemia-reperfusion, and the degree of atrophy was further exacerbated at 72 h. This suggests that skeletal muscle atrophy in the absence of IPC progresses gradually with time after ischemia-reperfusion. Conversely, IPC was observed to significantly inhibit skeletal muscle atrophy after 48 h. Therefore, it was suggested that the use of IPC can enable early recovery and commencement of rehabilitation for skeletal muscle that was used as the tourniquet for a long period.

Furthermore, it has also been shown that inflammatory cytokines are released from damaged cells. Inflammation generated by tissue failure may cause further tissue damage and adversely affect tissue repair. Cai et al. reported that IPC induced myocardial protective signals by suppressing inflammatory cytokines through ischemia-reperfusion injury in the myocardium [17]. In this experiment, COX-2, an inflammatory marker, was examined to confirm the anti-inflammatory effect of IPC. The IPC (−) group showed the infiltration of inflammatory cells and increased COX-2 expression, indicating strong inflammation induced by ischemia-reperfusion. However, the IPC (+) group confirmed the infiltration of inflammatory cells and suppression of COX2 expression. Therefore, with the increased VEGF expression by IPC, it was thought to reduce the inflammatory response after ischemia-reperfusion.

IPC preconditioning may suppress irreversible skeletal muscle injuries that occur when tourniquets are used for a long time. Additionally, as a mechanism for suppressing skeletal muscle injury caused by IPC, it is thought that the increased VEGF expression may reduce oxidative DNA damage after ischemia-reperfusion and lead to attenuation of the inflammatory response. Since ischemia caused by IPC is very short and adverse effects on skeletal muscle are unlikely, it is very useful because it can be applied to limb surgery using a tourniquet relatively easily. This study has a limitation. Tissue disorders such as apoptosis or necrosis that occurred in this study were not evaluated in functional aspects such as muscle strength, gait, and movement. Additionally, since the decreased effect of IPC due to age and comorbidities such as hypertension has been reported, it is necessary to add functional evaluations in the future, to examine them by spontaneously hypertensive rats and other factors, such as diabetes [18,19,20] or hypercholesterolemia, and to evaluate them at different ages.

## Data Availability

Data sharing is not applicable to this article as no datasets were generated or analyzed during the current study.
